# RGB-D Image Processing Algorithm for Target Recognition and Pose Estimation of Visual Servo System

**DOI:** 10.3390/s20020430

**Published:** 2020-01-12

**Authors:** Shipeng Li, Di Li, Chunhua Zhang, Jiafu Wan, Mingyou Xie

**Affiliations:** School of Mechanical and Automotive Engineering, South China University of Technology; Guangzhou 510641, China; lsponline@163.com (S.L.); itdili@scut.edu.cn (D.L.); mejwan@scut.edu.cn (J.W.); xmy3557@163.com (M.X.)

**Keywords:** RGB-D sensor, visual servoing, target recognition, depth measurement, pose estimation

## Abstract

This paper studies the control performance of visual servoing system under the planar camera and RGB-D cameras, the contribution of this paper is through rapid identification of target RGB-D images and precise measurement of depth direction to strengthen the performance indicators of visual servoing system such as real time and accuracy, etc. Firstly, color images acquired by the RGB-D camera are segmented based on optimized normalized cuts. Next, the gray scale is restored according to the histogram feature of the target image. Then, the obtained 2D graphics depth information and the enhanced gray image information are distort merged to complete the target pose estimation based on the Hausdorff distance, and the current image pose is matched with the target image pose. The end angle and the speed of the robot are calculated to complete a control cycle and the process is iterated until the servo task is completed. Finally, the performance index of this control system based on proposed algorithm is tested about accuracy, real-time under position-based visual servoing system. The results demonstrate and validate that the RGB-D image processing algorithm proposed in this paper has the performance in the above aspects of the visual servoing system.

## 1. Introduction

In recent years, the use of robotic operations based on visual instructions in modern manufacturing has rapidly increased. Visual servoing as an active visual technology has attracted everyone’s attention. However, both of the two main visual servoing methods—position-based visual servoing (PBVS) and image-based visual servoing (IBVS) have bottlenecks that constrain the development of this field: After successfully identifying the target object, the PBVS needs to reconstruct the target object in three dimensions, while the IBVS needs to approximate the distance between the end of the arm and target image. This means that they have higher precision requirements on depth information of the target image in a camera field of view. Researchers have done a lot of work to resolve these issues [1,2], but there are still some unresolved problems: The single-camera based attitude estimation method cannot generally produce a transformation matrix with absolute scale. For example, the stereo attitude estimation method using the stereo disparity relationship to calculate the depth, requires at least two cameras, which increases the system costs in the low-cost development trend of modern smart factories [3,4]. In addition, this method is limited by the distance between the two cameras, if the distance is too small, estimation accuracy of the long-distance target is poor [5].

The RGB-D cameras (Leap-Motion, Kinect, RealSense, uSens, and ThisVR), have been applied in many fields due to their ability to capture both RGB images and depth information [6,7]. These features provide a reference for solving target depth estimation problem in visual servoing system [8]. For example, Yi et al. solved the cumulative position error and large calculation problem of the traditional algorithm based on wheeled mobile robot by using the depth ranging capability of RGB-D camera [9], but this method has a low image recognition and positioning accuracy. Celine et al. used the dense depth map obtained from a range sensor as a visual feature for positioning task for nonplanar scenes, with no feature extraction or matching step [10], also he used an impossible recognition target which is smaller than millimeters. TSAI et al. proposed a 3D object recognition and attitude estimation algorithm based on RGB-D image, which converts the input RGB-D image into color point cloud data and extracts scene features from the color point cloud [11], however, this method has a poor effect when the target backgrounds are similar, and also has high requirements on image processing hardware. Lin et al. proposed a RGB-D vision control system comprising of a visual perception module, a target pose estimation module, a data argument module, and a robotic robot controller, this system is mainly used to improve the success rate for scene segmentation problems, but the method is only about 80% effective in model-based attitude estimation, and the experimental scene is strict, which is significantly affected by light and vibration [7]. Guo et al. used depth learning technology based on RGB-D camera to estimate target pose and proposed a custom loss function for the constraint function of parameter in pose estimation, however, the effect on the accuracy of pose estimation was not covered. As mentioned-above, the RGB-D sensor not only has high target recognition rate in a complicated environment [11], but also can accurately estimate the 3D attitude information of the object relative to the camera. Merely, according to the special role of the cameras in visual servoing system, there are still few image processing algorithms based on RGB-D cameras appearing in the field of visual servoing.

Benefiting from the above research and the application research ideas of RGB-D sensor in other fields [12,13], and insufficient in the existing visual servo algorithm for robots using RGB-D cameras as sensors, this paper proposes an effective image processing algorithm based on RGB-D sensor for visual servo research topics. Generally speaking, the traditional visual servoing system uses the industrial camera that is required to estimate the pixel depth of the image at each moment, but the proposed image algorithm can use the RGB-D sensor to capture the color image in real time and the depth information of each pixel of the image. The distortion coefficients of the two camera module images are combined to achieve the control purpose of PBVS under RGB-D camera.

The composition of this paper is as follows: Section 1 reviews the research status of image recognition and 3D reconstruction based on RGB-D camera; Section 2 provides the system structure of the target recognition and poses the estimation algorithm proposed in this paper; Section 3 introduces the theoretical basis and the partial derivation process of target recognition and 3D pose estimation, also the control law that is adopted in this paper; Section 4 presents the experiments to validate the effectiveness of the proposed image processing algorithm; Section 5 summarizes the experimental results of this paper and proposes solutions to the shortcomings and defects.

## 2. System Structure

**Figure 1:**
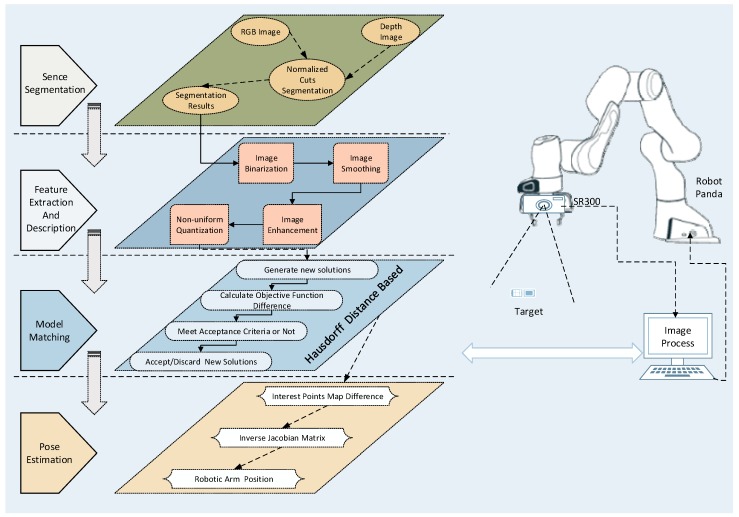
Visual servo control structure and image processing composition.

The system structure of the proposed platform is shown in Figure 1. At the beginning, the color and the depth images of scene are acquired by RGB-D camera, and then the color image is subjected to scene segmentation, feature extraction, and gray scale reduction. Next, the object recognition module detects the foreground object in the scene and records it in the database. The feature descriptors are matched with the target feature model to identify the objects of interest. The 3D reconstruction of the target image is completed by combining the distortion coefficients of image pixel depth information. Lastly, the current image is matched with the target image to complete the servo process. During this process, the manipulator performs inverse kinematics calculation based on the difference between current and target images to obtain the angle and the speed of the robot. The angle and the speed commands are then used to control the arm of the robot by an iterative cycle. The servo process utilizes the depth ranging module of the RGB-D camera to improve the depth estimation accuracy, optimize the image processing speed, and increase the system stability.

## 3. Target Recognition

This section describes the processing steps of the proposed RGB-D sensor-based 3D object recognition and matching module that is mainly composed of five unit modules: Scene segmentation, feature extraction and description, 3D model matching, pose estimation, and control law of visual servoing. After acquiring and binarizing the color image, the scene segmentation unit is responsible for removing the background point from binarization image. Then, the feature description unit is used to construct the feature descriptor of all foreground objects in the scene. Lastly, the descriptor matches the fused depth ranging unit information, completes the 3D reconstruction of current target object, and matches target pose.

### 3.1. Scene Segmentation

Scene segmentation is the primary task of target detection and recognition. The purpose of this task is to separate the foreground target and the planar background area of the scene in order to simplify or change the representation of scene, make the understanding and the recognition of the scene easier, and improve the computational efficiency in the subsequent feature extraction and description process. It has a wide range of applications in scene recognition, positioning, and even mapping of industrial robots [14,15]. In recent years, a large number of methods have been proposed for image segmentation through images, such as graph theory-based methods [16], clustering algorithm-based methods [17], digital image watermarking [15], and watershed transformations [18]. However, these methods are not ideal for segmenting the objects with similar colors or having the same color as the background in the scene. The reason for this is that the depth of field information is lost when the 3D scene is projected onto the 2D planar, so the information obtained from the single image is insufficient.

In this paper, considering the requirements of visual servoing system for estimating image depth information, the working environment of industrial scene camera is restored to the maximum extent, and the camera depth module is adopted to obtain the depth information of target image in the scene.

**Figure 2:**
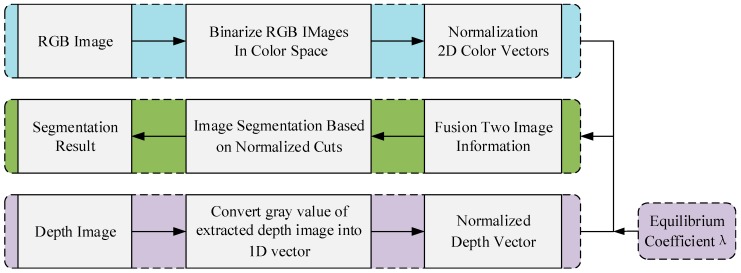
Composition of the proposed scene segmentation algorithm of RGB-D image.

The proposed scene segmentation algorithm is shown in Figure 2. Suppose a general scene **S** is composed of **n** points P(i=1,2,⋯,n) characterized by color and depth information. First, the color and the depth information need to be unified in an efficient way. In addition, a uniform color space will make the distances in each color component comparable, thus simplifying the clustering process of 3D vectors related to the color information. In this paper, RGB space is selected as the expression space of the color information, that is, the color information of each scene point p(i=1,2,⋯,n) is represented by a 3D vector as:

(1) $$p_{i}^{c}=\left[\begin{array}{c} R\left(p_{i}\right)\\ G\left(p_{i}\right)\\ B\left(p_{i}\right) \end{array}\right]\;\;i=1,2,\cdots ,n$$

Since the gray value of depth map is proportional to the distance of actual object from the depth camera, the depth information can be simply represented by the gray value of each point $p_{i}\in S$ in depth map. As the depth map is used in this paper, depth information is represented by the vector shown in Equation (2):

(2) $$p_{i}^{g}=\left[z\left(p_{i}\right)\right]\;i=1,2,\cdots ,n$$

As the scene acquisition system provides the relative distance of the scene depth instead of absolute distance, so the ideal scene segmentation algorithm should be insensitive to the relative scaling of depth information described by point cloud or depth map. In order to ensure that the segmentation algorithm is independent of depth image scaling, the depth vector needs to be normalized by the standard deviation of the scene point depth data. Additionally, final depth description is a vector as shown in Equation (3):

(3) $$\bar{z}\left(p_{i}\right)=\frac{1}{\sigma_{g}}\left[z\left(p_{i}\right)\right]$$

$\bar{z}\left(p_{i}\right)$ represents the normalized depth vector, is a key parameter in visual servo jacobian matrix estimation, the derivation of this parameter is visible in [19]. In order to balance the correlation of the two types of information (color and depth) in the merging process, the color information vector in Equation (1) is normalized by the standard deviation average of the three components *R*, *G*, and *B* corresponding to color information. In addition, final color information is described as:

(4) $$\left[\begin{array}{c} \bar{R}\left(p_{i}\right)\\ \bar{G}\left(p_{i}\right)\\ \bar{B}\left(p_{i}\right) \end{array}\right]=\frac{3}{\sigma_{R}+\sigma_{G}+\sigma_{B}}\left[\begin{array}{c} R\left(p_{i}\right)\\ G\left(p_{i}\right)\\ B\left(p_{i}\right) \end{array}\right]=\frac{3}{\sigma_{c}}\left[\begin{array}{c} R\left(p_{i}\right)\\ G\left(p_{i}\right)\\ B\left(p_{i}\right) \end{array}\right]$$

**Figure 3:**
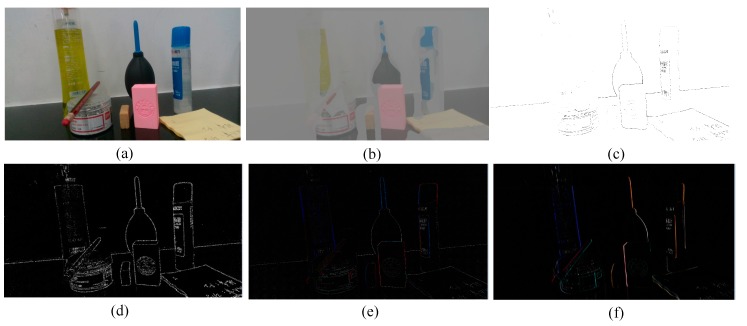
Scene segmentation graphs under different algorithms. (**a**) Original image; (**b**) three-dimensional (3D) segmentation; (**c**) automatic threshold segmentation; (**d**) Harris segmentation; (**e**) Laplace segmentation; (**f**) Sobel segmentation.

**Figure 4:**
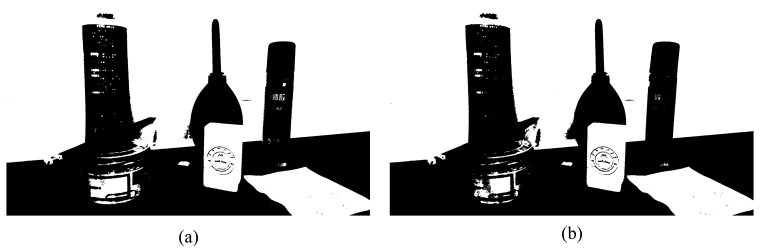
Different binarization segmentation results. (**a**) Conventional binarization segmentation; (**b**) binarization segmentation based on normalized cuts.

From the above normalized depth and color information vectors, representation of each scene point can be obtained as:

(5) $$p_{i}^{f}\left[\begin{array}{c} \bar{R}\left(p_{i}\right)\\ \bar{G}\left(p_{i}\right)\\ \bar{B}\left(p_{i}\right)\\ \lambda \bar{z}\left(p_{i}\right) \end{array}\right]\;\;i=1,2,\cdots ,n$$

where *λ* is a parameter that balances the color and the depth information, in the experiment part, for best results and based on the experience we adopt λ=1. This paper only describes the scene briefly due to space limitation. The detail description of the normalized cuts algorithm based on original image segmentation obtained by the RGB-D sensor is available in [20]. Figure 3 shows the image segmentation results for the same scene obtained using different algorithms. Where a is the original image, Figure 3b–3f are the image processing results of traditional segmentation algorithms. Figure 4a,b is the binarized images obtained using the conventional algorithm and the clustering algorithm in this paper, respectively. By comparing the segmentation results under the algorithms of Figure 3 and Figure 4, it can be seen that the binarization algorithm based on normalized cuts can separate the target object from other objects using the clustering method, and can effectively reduce the interference noise around the target object. After completing the segmentation based on normalized cuts, the feature values of the target object are extracted, and the grayscale image is restored when the target feature value is unchanged, and the target recognition is performed on this basis.

### 3.2. Feature Extraction and Description

**Figure 5:**
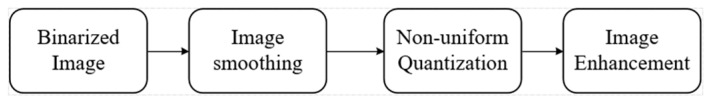
Image processing flow after binarized.

After completing the image segmentation task, the information of the image needs to be further processed: Such as image denoising, color correction, signal to noise ratio improvement, smoothing, sharpening, positioning, and separation. Restore or reconstruct the degraded images, improve the image fidelity, and provide clear images for viewers. In terms of image processing content, two problems must be solved: First one is to determine whether there is information needed in the image, and another one is to determine the information required to further extract and identify the image features. Based on the characteristics of binarized images shown in Figure 1, the processing flow is employed in Figure 5.

Image enhancement module is followed.

Image enhancement refers to the processing of highlighting certain information in an image according to specific requirements, while reducing or removing some unnecessary information. The purpose is to improve the image quality and enhance the recognition ability of certain information in the image for analysis and use [7]. Currently, several methods are available for image enhancement, which can be divided into two major categories: Spatial domain methods and frequency domain methods. The steps of the image enhancement algorithm used in this paper are as follows:

First, a histogram of the target image is constructed.

Histogram is a statistical graph for expressing the gray scale distribution of an image. It consists of where the abscissa is the gray value r and the ordinate is the gray value probability density p(r).

(6) $$p\left(r_{i}\right)=\frac{Number\;of\;pixels\;with\;gray\;value\;r_{i}}{Total\;number\;pixels\;of\;the\;image}\;\left(i=0,1,\cdots ,n\right)$$

And

(7) $$\overset{k-1}{\underset{i=n}{\sum }}p\left(r_{i}\right)=1$$

Then, the image histogram is equalized.

Let the total number of pixels of the original image be N, with L(L=256) gray levels, and frequency of i gray level $r_{i}$ is $n_{i}$, if the original image of pixel gradation at point (i, j) is $r_{i}$, the gradation of the histogram equalized image at point (i, j) is:

(8) $$S_{i}=T\left[r_{i}\right]=\overset{k}{\underset{i=1}{\sum }}\frac{n_{i}}{N}=\overset{k}{\underset{i=1}{\sum }}p_{r}\left(r_{i}\right)$$

The gray value after histogram equalization is inversely transformed into the original image, and the gray value of each pixel point is reset to reconstruct the final binarized image.

**Figure 6:**
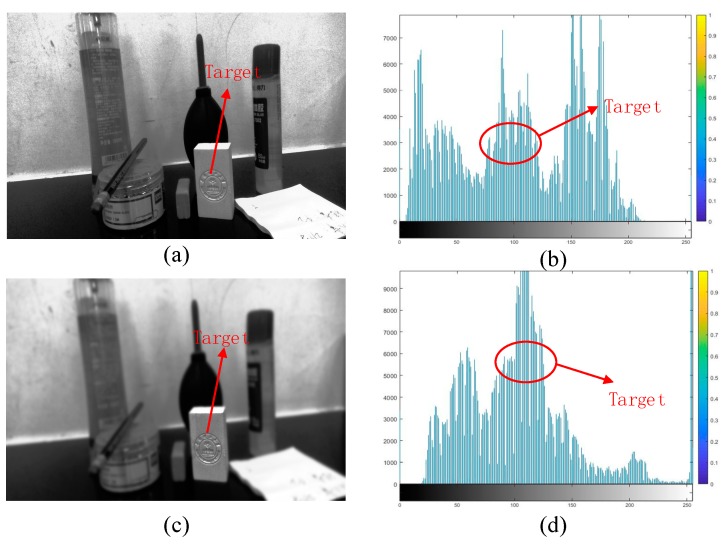
Restore grayscale images and their equalized images. (**a**,**b**) Shows target original gray scale image and its histogram; (**c**,**d**) shows after equalized the target image and its histogram.

Image processing results are as shown in Figure 6, where (a) is the original gray scale image and its histogram, while (b) is the image after equalizing the target image and its histogram. It can be seen from the figure that equalized histogram effectively highlights the target image information in the environment, and at the same time reduces the unnecessary image information around the periphery, which improves the target image quality and enhances the ability of recognizing the interest points in the image.

### 3.3. Model Matching

After extracting the interest image based on the image algorithm of RGB-D camera, a description matching process needs to be performed in order to find the 3D correspondence between the detected model object and the recorded model. The way to realize the precise positioning of moving objects with known 3D models using the monocular gray image is an important issue in the field of machine vision and a primary task of visual detection and target tracking based on 3D model. The key to its positioning lies in establishing a correspondence relationship between the target image and the model [21].

The Hausdorff distance can be used to measure the degree of matching between two sets of points. Since it does not need to establish an exact point-to-point correspondence between the model and the image, it is more fault tolerant than other matching methods [22]. In this section, the simple and feasible boundary extraction is used to filter out the influence of noise points from the boundary on the model matching by MHD (modified Hausdorff distance) algorithm. At the same time, in order to avoid looking for visible point in image and model projection pixel points, the displacement and the rotation of the model are directly defined in 3D space, and a global matching optimization function between target image and 3D model planar projection is established with the coordinate transformation and the Hausdorff distance. Finally, simulated annealing algorithm is used to obtain the optimal solution of the pose to improve the accuracy of matching.

The derivation process of matching optimization function based on Hausdorff distance is described below. The establishment of model and solution of matching parameters can be found in [21]. Let $T=\left\{t_{j}|1\leq j\leq I\right\}$ be the boundary contour point set extracted from the target interest point, $M=\left\{m_{i}|1\leq i\leq L\right\}$ be the line segment collection for wireframe models, and $R=R_{2}R_{1}R_{0}$ is the transformation matrix from model coordinates to image coordinates. The shape matching based on Hausdorff distance can be expressed as:

(9) $$\min\left(H\left(R\left(M\right),T\right)\right)=\min\left(H\left(\hat{M},T\right)\right)$$

where $\hat{M}$ is the projection of M in the image coordinate system, $\hat{M}=\left\{{\hat{m}}_{i}|1\leq i\leq L\right\}$, following is the simple derivation:

$$H\left(\hat{M},T\right)=\max\left(h\left(\hat{M},T\right),h\left(T,\hat{M}\right)\right)$$

$$h\left(T,\hat{M}\right)=\frac{1}{\sum_{t_{j}\in T}W_{t_{j}}}\underset{t_{j}\in T}{\sum }W_{t_{j}}\cdot \underset{{\hat{m}}_{i}\in \hat{M}}{\min}d\left(t_{j},{\hat{m}}_{i}\right)$$

(10) $$h\left(\hat{M},T\right)=\frac{1}{\sum_{{\hat{m}}_{i}\in \hat{M}}W_{{\hat{m}}_{i}}}\underset{{\hat{m}}_{i}\in \hat{M}}{\sum }W_{{\hat{m}}_{i}}\cdot \underset{t_{j}\in T}{\min}d\left(t_{j},{\hat{m}}_{i}\right)=\frac{1}{L}\underset{{\hat{m}}_{i\in }\hat{M}}{\sum }\underset{t_{j}\in T}{\min}d\left(t_{j},{\hat{m}}_{i}\right)$$

In the above formula, $d\left(t_{j},{\hat{m}}_{i}\right)$ is defined as the distance from point $t_{j}$ to line segment $m_{j}$. Given point $t_{j}$ and line segment $m_{j}$ (determined by endpoints $b_{1}$ and $b_{2}$), then

(11) $$\left(t_{j},{\hat{m}}_{i}\right)=\{\begin{array}{c} \left|\bar{t_{j}{\hat{t}}_{j}}\right|\;if\;\bar{t_{j}b_{j1}}\cdot \bar{b_{1}b_{2}}\;>0\;\\ else\;\\ \underset{b\in \left(b_{1},b_{2}\right)}{\min}\left|\bar{t_{j}b}\right| \end{array}$$

**Figure 7:**
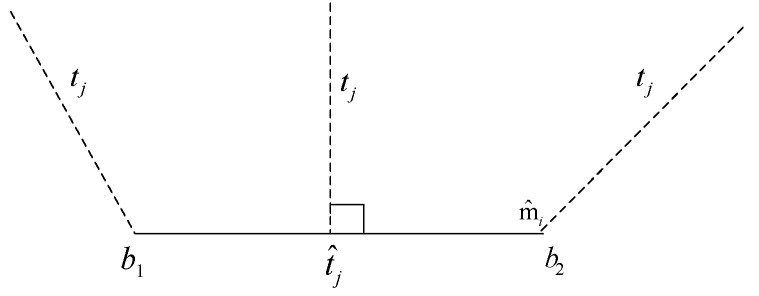
Distance from point $t_{j}$ to line $m_{j}$ under different conditions.

**Figure 8:**
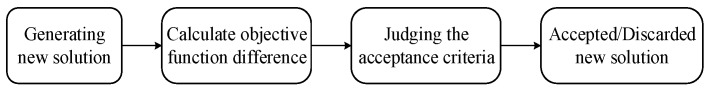
Simulated optimal annealing algorithm calculation process.

As shown in Figure 7:

The figure above is a visual representation of formula (11), shows the distance from point $t_{j}$ to line $m_{j}$ under different conditions. As shown above from Equations (9)–(11), the objective function formula (9) is a continuous nondifferentiable multivariable function and there are multiple local minimum points in search space, it should be noted that in the actual calculation process, some parameters involved in the formula can be given according to the situation to reduce unnecessary complicated operations. In this paper, the simulated optimal annealing algorithm is used to obtain the global optimal solution of the objective function the algorithm is simple, versatile, robust, and suitable for parallel processing. The calculation process is a series of iterative processes, as shown in Figure 8.

In order to avoid duplication of content, matching results of the model are presented in the experiment section.

### 3.4. Pose Estimation

In the visual servoing system of this paper, RGB-D camera or planar camera is fixed at the end of the robot arm. After capturing the current image, it then needs to be matched with the target image. The angle and the speed of the robot arm are obtained by inverse kinematics, which requires a composite Jacobian matrix that correlates the speed of the robot and the image change. Inspired by the registration of point cloud images [23], this section uses the RANSAC (random sample consensus) algorithm to provide an initial transformation matrix for RGB-D image fine registration. The RANSAC is an iterative algorithm for estimating the parameters of mathematical models, which are used to determine the corresponding points that satisfy a particular mathematical model, and obtain the initial transformation matrix accordingly. After applying the initial transformation matrix to RGB-D images, the LM-ICP is applied to the transformed RGB-D images and scenes, which is an improved version of the ICP algorithm. A common optimization algorithm for nonlinear optimization of objective functions is the least squares optimization algorithm. The final solution can be obtained using the SVD algorithm [24]. However, all norm optimization problems are sensitive to discrete points due to square of residuals. Therefore, the Huber loss function is used as a cost function for the corresponding point set:

$$p=\left\{p_{0},p_{1},p_{2},\cdots ,p_{n-1},p_{n}\right\},\;q=\left\{q_{0},q_{1},q_{2},\cdots ,q_{n-1},q_{n}\right\}$$

(12) $$e^{2}\left(n\right)=\{\begin{array}{ll} n^{2}/2 & \left|n\right|\leq k\\ k\left|n\right|-n^{2}/2 & \left|n\right|\geq k \end{array}$$

where $n=\left\|(R\cdot p-t\right)-q{\|}_{2}$ is the distance between the corresponding points and k is the distance threshold, the Huber loss function is smooth and differentiable here. Similar to the objective function of point cloud registration, the expression is:

(13) $$\hat{R},\hat{t}=arg\underset{R,t}{\min}\overset{N}{\underset{i=1}{\sum }}e^{2}\left(n\right)$$

Get the optimal $\hat{R},\hat{t}$ by the LM-ICP algorithm [25], if (α,β,γ) and $\left(t_{x},\;t_{y},\;t_{z}\right)$ are the three rotation angle translation components of the coordinate axis, respectively, the end of the 6 DOF manipulator can be expressed as $\left(\alpha ,\beta ,\gamma ,t_{x},\;t_{y},\;t_{z}\right)$, and the optimal transformation matrix can be expressed as:

(14) $$M=T\left(t_{x},\;t_{y},\;t_{z}\right)\cdot R\left(\alpha ,\beta ,\gamma \right)$$

(15) $$T\left(t_{x},\;t_{y},\;t_{z}\right)=\left[\begin{array}{c} \begin{array}{cc} 1 & \begin{array}{ccc} 0 & 0 & t_{x} \end{array} \end{array}\\ \begin{array}{ccc} 0 & 1 & \begin{array}{cc} 0 & t_{y} \end{array} \end{array}\\ \begin{array}{ccc} 0 & 0 & \begin{array}{cc} 1 & t_{z} \end{array} \end{array}\\ \begin{array}{ccc} 0 & 0 & \begin{array}{cc} 0 & 1 \end{array} \end{array} \end{array}\right]$$

(16) $$\begin{array}{ll} R\left(\alpha ,\beta ,\gamma \right)=R_{z}\left(\gamma \right)\cdot R_{y}\left(\beta \right)\cdot R_{x} & =\left[\begin{array}{cccc} r_{11} & r_{12} & r_{13} & 0\\ r_{21} & r_{22} & r_{23} & 0\\ r_{31} & r_{32} & r_{33} & 0\\ 0 & 0 & 0 & 1 \end{array}\right]\\ & =[\begin{array}{cccc} \cos\gamma \cos\beta & -\sin\gamma \cos\alpha +\cos\gamma \sin\beta \sin\alpha & \sin\gamma \sin\alpha +\cos\gamma \sin\beta \cos\alpha & 0\\ \sin\gamma \cos\beta & \cos\gamma \cos\alpha +\sin\gamma \sin\beta \sin\alpha & \cos\gamma \sin\alpha +\sin\gamma \sin\beta \cos\alpha & 0\\ r-\cos\beta & \cos\beta \sin\alpha & \cos\beta \cos\alpha & 0\\ 0 & 0 & 0 & 1 \end{array}] \end{array}$$

It can be derived

(17) $$(\alpha ,\beta ,\gamma )=(\arctan\left(r_{32}/r_{33}\right),\arcsin\left(-r_{31}\right),\arctan\left(r_{21}/r_{11}\right)).$$

As mentioned above, α,β,γ are the rotation angles of the three axes. Calibration of the hand-eye relationship between the camera and the robot are needed before experiment.

### 3.5. Control Law of Visual Servoing

The aim of all vision-based control schemes is to minimize an error e(t), which is typically defined by

(18) $$e\left(t\right)=s\left(m\left(t\right),a\right)-s^{*}$$

This control law comes from [19], the parameters in Equation (18) are defined as follows: The vector m(t), is a set of image measurements (e.g., the image coordinates of interest points or the image coordinates of the center of an object). These image measurements are used to compute a vector of k visual features, s(m(t),a), in which a is a set of parameters that represent potential additional knowledge about the system (e.g., coarse camera intrinsic parameters or 3D models of objects). The vector $s^{*}$ contains the desired values of the features is constant, and changes in s depend only on camera motion. Further, we consider here the case of controlling the motion of a camera with six degrees of freedom (6 DOF); e.g., a camera attached to the end effector of a six degree-of-freedom arm.

In this paper, we describe a position-based visual servo control (PBVS), which consists of a set of 3D parameters, which must be estimated from image measurements.

Once s is selected, the design of the control scheme can be quite simple. Then, we need to design a velocity controller, to solve this problem, we should know the relationship between the time variation of s and the camera velocity. Let the spatial velocity of the camera be denoted by $V_{c}=\left(v_{c},\omega_{c}\right)$, with $v_{c}$ the instantaneous linear velocity of the origin of the camera frame and $\omega_{c}$ the instantaneous angular velocity of the camera frame. The relationship between $\dot{s}$ and $v_{c}$ is given by:

(19) $$\dot{s}=L_{s}v_{c}$$

in which $L_{s}$ ∈ $R^{k\times 6}$ is named the interaction matrix related to s. The term feature Jacobian is also used somewhat interchangeably in the visual servo literature.

Using (18) and (19), we can get the relationship between camera velocity and the time variation of the error:

(20) $$\dot{e}=L_{e}v_{c}$$

where $L_{e}=L_{s}$. Considering $v_{c}$ as the input to the robot controller, and if we would like, for instance, to try to ensure an exponential decoupled decrease of the error (i.e., $\dot{e}=-\lambda e$), we obtain using (20):

(21) $$v_{c}=-\lambda L_{e}^{+}e$$

where $L_{e}^{+}$ ∈ $R^{6\times k÷}$ is chosen as the Moore-Penrose pseudoinverse of $L_{e}$.

In the next experiment part, the computed velocity of image Jacobian is $v_{c}$.

## 4. Experiment Results

**Figure 9:**
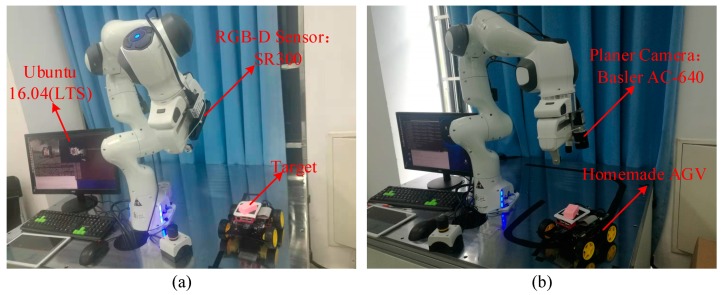
Experimental system composition. (**a**) Experimental system with RGB-D camera; (**b**) experimental system with planar camera.

In order to verify the effectiveness of the proposed RGB-D image processing algorithm in visual servoing system, this section uses an Intel Realsense SR300 (hereinafter referred to as SR300) camera as RGB-D sensor. First, the performances of the traditional camera and the RGB-D camera in target recognition and pose estimation under the visual servoing system are compared. Then, based on SR300 camera, the image algorithm proposed in this paper is compared with the general processing algorithm [10] in terms of system convergence speed and accuracy. The entire experiment contains three aspects: 1. Through the ordinary planar camera image algorithm and the image processing algorithm proposed in this paper, change of the manipulator translation error and rotation error in the visual servo process are used to illustrate the effect of RGB-D algorithm on servo convergence accuracy and speed; 2. The improved RGB-D image processing algorithm based on SR300 is compared with the traditional RGB-D image processing algorithm, which shows the improvement of image recognition accuracy and reduces the image processing load; 3. Based on the improved RGB-D image processing algorithm proposed in this paper, static and dynamic targets are tracked and tested, respectively. In order to effectively illustrate the effective recognition and extraction matching ability of the proposed image algorithm in the nonstructural environment, the object with low contrast is selected as the target of interest. The experimental system is shown in Figure 9.

In the experimental system: All the parameters involved in this experiment are set according to the previous description. The robot is named Panda produced by Franka Emika; the RGB-D Sensor is Intel RealSense SR300 camera, connected to the image processing system via USB3.0 and the planar camera is a Basler AC-640 connected to the image processing system via a Gige network. The interest target is a pink rectangular wooden block; mobile target carrier AGV is a homemade car based on Raspberry Pi; the image processing platform is a Linux 16.04 LTS operating system based on PREEMPT_RT kernel, equipped with Intel(R) Core (TM) i7-8700 CPU and 8 GB installed memory. Figure 9a is a visual servoing system based on Intel SR300, and Figure 9b is a visual servoing system based on Basler AC-640. The results are presented as follows.

### 4.1. Comparison between Planar Camera Algorithm and Proposed RGB-D Algorithm

**Figure 10:**
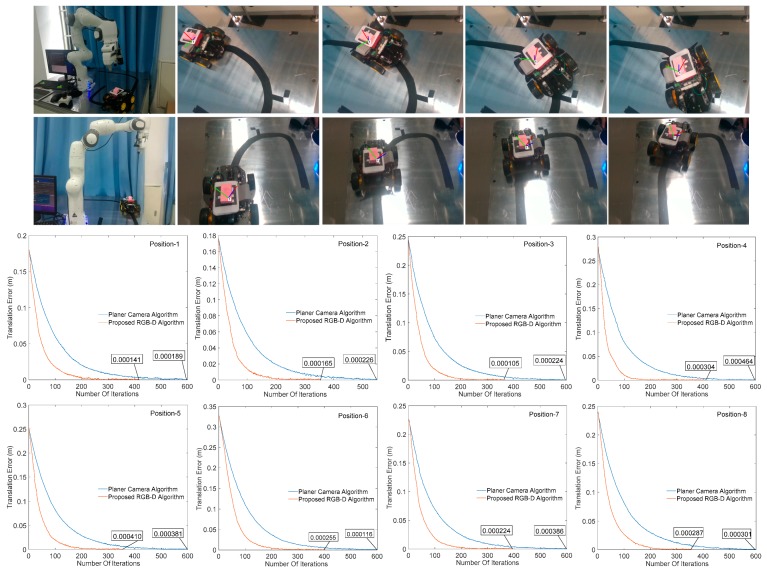
Change of manipulator translation error in different positions.

**Figure 11:**
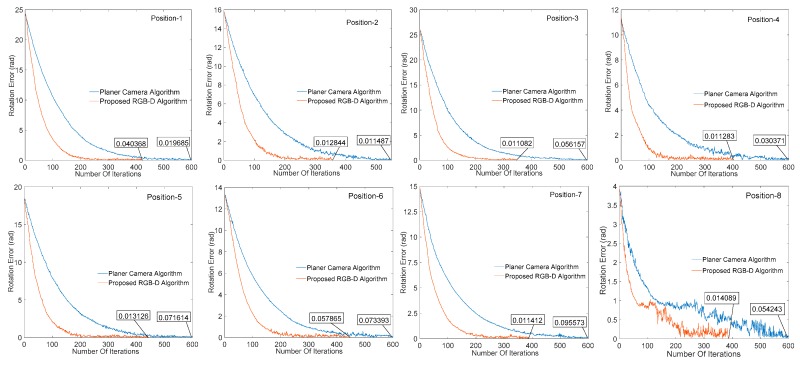
Change of manipulator rotation error in different positions.

In order to test the effectiveness of our proposed algorithm, the effects of image processing results on the convergence of visual servo system are illustrated by different cameras under different algorithms. Figure 10 and Figure 11 respectively show the change of the translation and rotation direction errors of the manipulator at different initial positions under the planar camera (AC-640) algorithm and the RGB-D (SR300) image processing algorithm proposed herein, wherein the abscissa is the number of iterations performed by the servo process, the ordinate is the translation change, and the rotation change value of the corresponding iterations, the unit is in m/t and rad/t (t represents an iteration cycle). It can be seen from Figure 10 and Figure 11 that all the eight positions results show that the RGB-D algorithm proposed in this paper can effectively increase the image processing speed and reduce the servo cycle time (planar algorithm convergence around 600 times, and our algorithm is around 350–400 times) without affecting the system convergence accuracy.

### 4.2. Comparison between Traditional RGB-D Algorithm and Proposed RGB-D Algorithm

**Figure 12:**
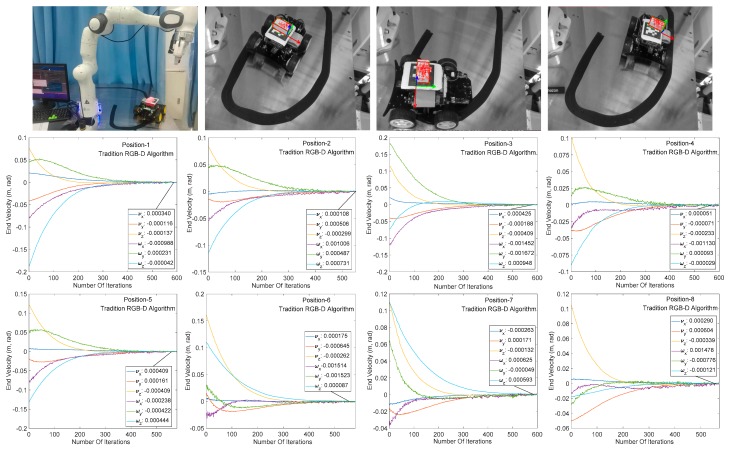
Manipulator speed change of traditional RGB-D image processing algorithm.

This section mainly compares the performance of the traditional RGB-D camera-based image algorithm with the SR300-based image algorithm in the visual servoing system. As shown in Figure 12, the RGB-D camera successfully identifies the target under both algorithms, completes the image processing process such as 3D modeling and pose estimation, and performs a visual servo experiment under the same initial and target positions.

**Figure 13:**
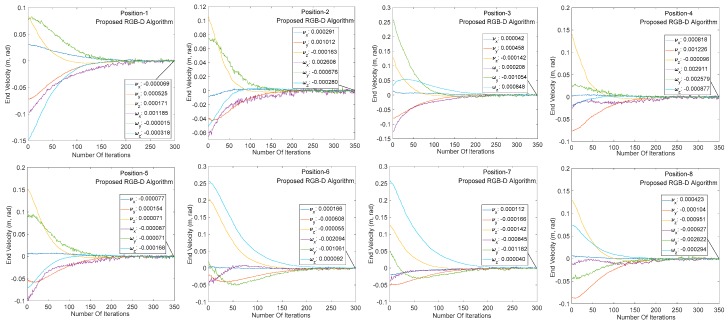
Manipulator speed change of proposed RGB-D image processing algorithm.

Figure 12 and Figure 13 show the end speed changes of the robot in the same servo task under the traditional algorithm and the proposed algorithm, respectively. The abscissa indicates the number of system servo iterations, and ordinate indicates the robot end speed change. It can be seen from the abscissa of the two graphs that the traditional RGB-D camera-based servo system converges between 500 and 600 iterations (single iteration is around 55 ms) at the same target distance. The iterative convergence of the servo system based on SR300 camera proposed in this paper is about 300 iterations (about 30 ms in a single iteration). In addition, the speed changes rapidly at beginning of the servo task, indicating that the image processing algorithm based on SR300 camera can effectively reduce the image processing time, reduce the computer load, and significantly reduce the system convergence time (to facilitate the display of speed change smoothness, reduce the initial image, and target image pose difference and convergence threshold). It can be seen from the speed changes of the arm in Figure 12 and Figure 13. The servo system has the same convergence precision under the two RGB-D image algorithms, which indicates the high precision and high speed properties of the proposed image algorithm in the visual servo system.

### 4.3. Effect of Image Algorithm on Visual Servoing Performance

In this section, the traditional industrial camera is replaced by the RGB-D camera SR300 in order to obtain a convenient, accurate, and economical visual servoing control system. It is necessary to verify the overall performance of the visual servoing system using RGB-D camera as sensor, in the meantime the image error change under the planar camera is provided. The experimental hardware part is shown in Figure 9. The following is an eye-in-hand (camera fixed at end of the arm) PBVS system, in which the identification matching of the static target and the recognition and the tracking effects of the dynamic target object are respectively verified.

**Figure 14:**
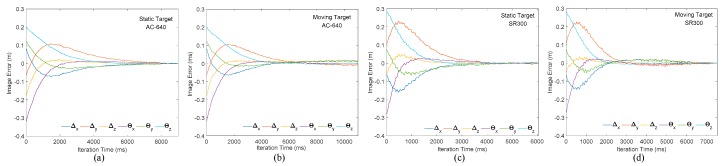
Image changes of the robot arm under planar and RGB-D cameras. (**a**,**b**) Represents the variation process of the robotic arm’s static and moving target matching error under the planar camera algorithm; (**c**,**d**) represents the variation process of the robotic arm’s static target and moving matching error under the proposed camera algorithm.

Figure 14a,b are the static target and dynamic target image changes under planar camera, respectively. $\Delta_{x},\Delta_{y},\Delta_{z}$ represents translation image error, $\theta_{x},\theta_{y},\theta_{z}$ represents rotation image error. It can be seen from Figure 14 that the system successfully converges under static target. However, under dynamic target, the robot arm always follows the target moving, because its iteration period is long and the convergence speed is slow, finally fails to achieve system convergence. Figure 14c is identification and tracking results of static target by the visual servoing system with SR300 as sensor. It can be seen from Figure 14c that the RGB-D camera guides the robot arm slowly according to inverse kinematics algorithm after effectively identifying the target object. The initial position is close to the target object and then adjusted after reaching the set depth position. Finally, the target position is reached under the guidance of the visual image. The convergence speed is obviously faster than that of the planar camera, which effectively validates the previous viewpoints of this paper. Figure 14d shows the robot arm tracking the moving target object under SR300. After the servo starts, the robot arm quickly tracks the moving target object (2500–6000 ms), and finally completes the servo convergence to the moving target, which again verifies the viewpoint of this paper.

## 5. Conclusions

In this paper, a new image recognition algorithm based on RGB-D camera is proposed. By improving the binarization algorithm based on normalized cuts, it is possible to effectively segment the scene in a complex environment, which simplifies the workload of 3D reconstruction and reduces the computer load (single visual servoing iteration time less than 30 ms). Model matching and pose estimation based on Hausdorff distance are realized by combining the target depth information after smoothing to enhance and restore the target image. The proposed RGB-D image processing algorithm can effectively improve the image matching efficiency, and the proposed image processing algorithm is applied in the PBVS system based on SR300 camera. The accuracy of the proposed algorithm is demonstrated by comparing with the performance of the traditional industry camera in manipulator translation and rotation error in the visual servoing system. Then, the traditional RGB-D image processing algorithm is compared with the proposed image processing algorithm under SR300 camera. The results demonstrate that the present optimization algorithm is characterized by image processing speed and system convergence precision. Finally, in the background of stationary and moving target tracing, the overall performance of the proposed RGB-D image algorithm and planar image algorithm in visual servoing system are verified and analyzed. The positive impact of the proposed RGB-D image algorithm on the accuracy, speed, etc., capability of the system is demonstrated.

Despite this, there are some points that still need to be improved during the RGB-D image processing. For example, when the target object or the interest point is too small, the environmental factors will have significant influence. Although, the target object can be effectively identified in a complex environment, if the environment filled with objects similar in color, shape, texture, or size, the image algorithm will be misjudged. Thus, the recognition accuracy needs to be improved. The accuracy is also related to the resolution of the RGB-D camera hardware. The response speed of the visual servo system is affected by camera frame rate, image processing algorithm, and system hardware configuration. The optimization of image processing and control algorithms and hardware upgrades (such as the introduction of cloud/fog/edge computing structures [4] in image processing) will further improve the system performance.
